# Pressure pain threshold and temporal summation in adults with episodic and persistent low back pain trajectories: a secondary analysis at baseline and after lumbar manipulation or sham

**DOI:** 10.1186/s12998-020-00326-5

**Published:** 2020-06-12

**Authors:** Sasha L. Aspinall, Angela Jacques, Charlotte Leboeuf-Yde, Sarah J. Etherington, Bruce F. Walker

**Affiliations:** 1 College of Science, Health, Engineering and Education, Murdoch University, 90 South St, Murdoch, WA 6150 Australia; 2 Institute of Regional Health Research, University of Southern Denmark, J. B. Winsløws Vej 19, 3, 5000 Odense C, Denmark

**Keywords:** Low back pain, Trajectories, Quantitative sensory testing, Spinal manipulation, Pressure pain threshold, temporal summation, Sensitization, Sensitisation, Hyperalgesia

## Abstract

**Background:**

People with chronic low back pain (LBP) typically have increased pain sensitivity compared to healthy controls, however its unknown if pain sensitivity differs based on LBP trajectory at baseline or after manual therapy interventions. We aimed to compare baseline pressure pain threshold (PPT) and temporal summation (TS) between people without LBP, with episodic LBP, and with persistent LBP, and to compare changes over time in PPT and TS after a lumbar spinal manipulation or sham manipulation in those with LBP.

**Methods:**

Participants were aged 18–59, with or without LBP. Those with LBP were categorised as having either episodic or persistent LBP. PPT and TS were tested at baseline. LBP participants then received a lumbar spinal manipulation or sham, after which PPT and TS were re-tested three times over 30 min. Generalised linear mixed models were used to analyse data.

**Results:**

One hundred participants (49 female) were included and analysed. There were 20 non-LBP participants (mean age 31 yrs), 23 episodic LBP (mean age 35 yrs), and 57 persistent LBP (mean age 37 yrs). There were no significant differences in PPT or TS between groups at baseline. There was a non-significant pattern of lower PPT (higher sensitivity) from the non-LBP group to the persistent LBP group at baseline, and high variability. Changes in PPT and TS after the interventions did not differ between the two LBP groups.

**Discussion:**

We found no differences between people with no LBP, episodic LBP, or persistent LBP in baseline PPT or TS. Changes in PPT and TS following a lumbar manual therapy intervention do not appear to differ between LBP trajectories.

**Trial registration:**

The trial was prospectively registered with ANZCTR (ACTRN12617001094369).

## Background

Low back pain (LBP) is associated with enormous disability and cost worldwide [1], and current treatment options tend to produce only modest positive outcomes. It is imperative that we improve our understanding of LBP, its causes, and contributing factors, which will hopefully pave the way for better prevention and management to reduce the burden of LBP.

Recent research on individuals’ experiences of non-specific LBP over time is leading away from the traditional model of LBP in three potential stages, acute, sub-acute, and chronic. Instead, LBP can be thought of as a life-long condition (like asthma) and described in terms of its pattern over time (called a trajectory) [2, 3]. Trajectory research makes use of frequent data collection about LBP symptoms over time. Two broad types of trajectories of LBP have emerged, episodic and persistent [4], with varying sub-categories such as fluctuating, mild persistent, and severe persistent [3]. There is evidence suggesting that an individual’s LBP trajectory tends to stay relatively stable over time [3, 5], supporting the clinical usefulness of trajectories to predict patient outcomes and plan management strategies. It has been suggested that the trajectories approach offers a more useful and nuanced framework for research and clinical decision making around LBP compared to the traditional model [3, 6].

Parallel to this, there has been significant interest in quantifying pain sensitisation processes in people with various painful conditions, including LBP, as pain sensitisation is implicated in the development of chronic pain [7]. This research has focused on comparing asymptomatic individuals to those with chronic pain, with pain sensitivity often assessed using quantitative sensory testing (QST). QST encompasses a variety of procedures for measuring participants’ responses to a standardised painful stimulus. We focus on pressure pain (detection) threshold (PPT) and temporal summation (TS), as this manuscript is a secondary analysis of a trial measuring only PPT and TS [8]. PPT is the threshold at which increasing pressure at a testing site becomes painful [9]. TS is a measure of how much the severity of a painful stimulus changes when the stimulus is repeated with three second intervals or less at a testing site [10]. Pain severity typically increases with repeated stimuli in healthy individuals [10], though responses are known to vary between individuals [11] and protocols [12]. TS is thought to assess the physiological phenomenon of wind-up, where dorsal horn neurons become increasingly excited in response to repeated noxious stimuli in a short time frame [10].

A recent systematic review concluded that PPT is decreased at remote sites in people with sub-acute and chronic LBP [13]. PPT is also decreased locally (at the lumbar spine) in people with sub-acute [14, 15] and chronic LBP compared to healthy controls [16–26]. TS measured with mechanical stimuli appears to be heightened at the lumbar spine but not the hand in people with chronic LBP [13].

Differences in QST between people with different LBP trajectories may provide a deeper understanding of sensitisation processes occurring in different clinical courses of LBP that is more graded than the traditional acute/chronic model. People with episodic LBP appear to have been overlooked as they typically won’t meet the criteria for chronic LBP used in many studies. Since people with episodic LBP experience pain-free periods of varying durations, they may at times be recruited into healthy control groups if eligibility criteria are not designed with episodic LBP in mind. Only a single study has compared QST in recurrent LBP vs. persistent LBP and healthy control groups; they found no significant differences in PPT or mechanical TS between any of these groups [27].

It has also been suggested that changes in QST outcomes after manual therapy interventions, such as spinal manipulative therapy (SMT) and mobilisation, may help explain the positive clinical outcomes for musculoskeletal pain seen in some patients [28]. Short-term increases in PPT and attenuation of TS have been observed following SMT and mobilisation in people with musculoskeletal pain [29, 30]. Since there are differences in pain processing and QST outcomes between asymptomatic and chronic LBP patients, and potentially between different LBP trajectories, it is also possible that changes in QST measures after an intervention may differ between LBP trajectories. To the best of our knowledge changes in QST based on LBP trajectories has not yet been investigated after any manual therapy interventions.

### Rationale and research questions

There is a paucity of prior research comparing baseline PPT and TS, and short-term changes in PPT and TS following lumbar manual therapy, in different LBP trajectories. We performed a planned secondary analysis of data collected during a clinical trial to investigate these gaps. Our research questions were as follows:

1. Are there baseline differences in PPT and TS between adults with no LBP, episodic LBP, and persistent LBP?
2. Are there differences in how PPT and TS change in the short term after a lumbar manual therapy intervention, between adults with episodic LBP and persistent LBP?

## Methods

This article uses a subset of data from a clinical trial with observational and randomised controlled trial components. We describe only the relevant methods and data in this manuscript. The trial was prospectively registered with ANZCTR (ACTRN12617001094369) and was approved by the Murdoch University Human Research Ethics Committee (approval 2017/177).

### Participants

Participants with and without LBP were recruited from the Murdoch University campus and the general public in Perth, Western Australia. Participants must have been aged 18 to 60 years. Participants were categorised as non-LBP participants if they agreed with the statement ‘I have NOT been bothered by LBP in the last 12 months,’ or as LBP participants if they agreed with the statement ‘I have been bothered by LBP at some time in the last 12 months.’ LBP participants were not required to have pain at the time of participating, and the concept ‘bothersome LBP’ has been used successfully in other studies [5, 31–33].

Exclusion criteria: a) contraindication to lumbar high-velocity low-amplitude SMT (since one intervention arm involved SMT), and b) any other condition that could alter QST measurements (upper or lower limb radiculopathy, neurological conditions, fibromyalgia, chronic widespread pain, or skin conditions at a QST testing site). Additionally, chiropractors and chiropractic students were excluded from participating as LBP participants, to reduce the possibility of expectancy bias and to improve blinding. Exclusion criteria were identified by self-report or on suspicion by the assessor based on a history and physical exam. We requested that participants avoid pain medications, recreational drugs, or moderate to heavy alcohol intake for 24-h prior, and to avoid chiropractic treatment for one week prior to participating.

### Procedure

All visits were conducted on the university campus in a temperature-controlled research room. After completing informed consent, LBP participants filled out a questionnaire on demographic information, LBP trajectory, LBP intensity, pain catastrophising, and anxiety. This was followed by a LBP history, and a physical examination including lumbar range of motion, lumbar orthopaedic tests, and lower limb motor-sensory examination, all performed by the assessor who is a chiropractor with 5 years’ clinical experience (SA). Non-LBP participants completed the same process except for the LBP intensity questions and LBP history.

Both non-LBP and LBP participants underwent baseline PPT and TS testing. Participants had at least two practice attempts for both the PPT and TS procedure on one forearm. They were then asked to lay prone on a treatment table. QST sites were marked bilaterally on the participants’ skin. Next, baseline QST was measured by the assessor, who had extensive experience testing PPT and TS. After this point, the non-LBP participants completed the study.

LBP participants were then randomly allocated to receive either: a) a single side-lying high-velocity low-amplitude SMT targeting the L5 segment on one side, or b) a sham lumbar manipulation in a similar side-lying position with a simulated thrust into the gluteal muscles, with the intention to deceive participants. Full information about the interventions and random allocation is published elsewhere [8]. The assessor and participants were blinded to group allocation, but the assessor was not blind to LBP trajectory group. Following the intervention, the assessor tested QST again immediately, and 15 and 30 min after the intervention. LBP participants were contacted by phone approximately 24 h following the visit in order to assess blinding. Of the LBP participants who received a sham manipulation, 62.5% thought they received a real treatment, suggesting that the sham was able to deceive the majority of LBP participants [8].

### Questionnaires

The Visual Trajectories Questionnaire-Pain contains visual and written descriptions of different back pain trajectories, and asks participants to select the trajectory that best matches their experience over a certain period of time [34]. We asked participants to select the trajectory that best represented their LBP experience over the previous year, with six options: a) “a single episode with no other major episodes of back pain,” b) “a few episodes of back pain with mostly pain-free periods in between,” c) “some back pain most of the time, and a few episodes of severe pain,” d) “pain that goes up and down all the time, with episodes of more severe back pain,” e) “severe back pain all or early all of the time,” and f) “no back pain, or only the odd day with mild pain” [34]. A previous study demonstrated the instrument has face validity, with the vast majority of participants finding the questionnaire acceptable and easy to answer [34]. For most participants in that study, the trajectories derived from the questionnaire and from frequent text messaging over six months matched, or were in a similar category, demonstrating acceptable criterion validity [34]. Participants with a more severe trajectory or more frequent episodes also had worse outcomes in other aspects of their health (e.g. pain radiation, disability, and psychological measures), indicating construct validity [34]. For our purposes, participants’ responses were collapsed into three trajectory groups as follows: a) and b) indicated episodic LBP, c) through e) indicated persistent LBP, and f) indicated non-LBP.

LBP intensity was measured in LBP participants only by asking them to rate their current LBP intensity, average LBP (when in pain) over the last 24 h, worst LBP in the last 24 h, and best LBP in the last 24 h. These were rated on 0 to 10 numerical rating scales (NRS, 0 = no pain and 10 = worst pain imaginable).

The Pain Catastrophizing Scale asks participants about 13 items relating to negative thoughts and feelings they may have experienced during painful events. Scored from zero to 52, a higher score indicates greater catastrophising. The scale has demonstrated internal consistency, construct validity, and the ability to discriminate between populations with clinical pain and from the community [35].

The Patient Reported Outcomes Measurement Information System (PROMIS®) Short Form v1.0 –Anxiety 6a involves questions about six feelings associated with anxiety over the last seven days. T-scores are reported here, which are based on the general United States population with a mean and standard deviation of 50 ± 10, with conversion from raw scores based on information available from PROMIS. Internal consistency between the short form and full-length questionnaire has been demonstrated [36], and the questionnaire has shown acceptable discriminative ability and responsiveness to change in clinical populations [37]. Good concurrent validity has also been demonstrated against a diagnostic anxiety questionnaire [38].

### Quantitative sensory testing

At each QST round, PPT testing was followed by TS testing, with each round taking roughly 10 min. PPT was measured three times at each testing site following a circuit [39] at the following locations bilaterally: a) mid-belly of the medial gastrocnemius, b) 2 cm adjacent to the L5 spinous process, and c) mid-belly of the middle deltoid. A calibrated digital pressure algometer (FPIX 50, Wagner Instruments, Connecticut, USA) with a circular 1cm^2^ rubber probe was used. The algometer was connected to a laptop via a cable, allowing measurements to be recorded electronically. Standard protocol was followed [9], with the assessor placing the probe perpendicularly to the skin and increasing the pressure at a rate of roughly 500 g/sec, while monitoring the force reading on the algometer. The participant was asked to indicate the moment the sensation of pressure first became painful by saying “Yes”, after which the assessor removed the algometer. For data analysis, the second and third measures were averaged, and right and left sides combined [40].

TS was also measured three times at each testing site, alternating between left and right, at the following locations bilaterally: a) middle of the proximal transverse arch of the palmar hand, and b) middle of the anterior transverse arch of the foot. TS was produced with a pinprick stimulus using the Neuropen with Neurotips (Owen-Mumford, Woodstock, UK). For each stimulus, the tip was pressed briefly into the testing site until markers on the device lined up. One stimulus was delivered first, and the participant was asked to verbally rate the severity of that stimulus. Then a series of five stimuli were delivered in a row at a rate of one per second at the same site (within a 1cm^2^ area of skin), and the participant was asked to rate the severity of the final stimulus. All ratings were on a 101-point NRS where 0 = no pain and 100 = worst pain imaginable. Our protocol differs from that defined by the German Research Network on Neuropathic Pain (DFNS) [41], as we were concerned about the ‘unpleasantness’ burden on LBP participants with repeated QST testing over a 2 h time period. We pre-tested both the pinprick device and protocol on ten asymptomatic participants, finding our protocol produced acceptable TS [42]. TS for each participant and each testing site was calculated by subtracting the mean first pinprick rating from the mean final pinprick rating, and averaging right and left sides.

### Statistical analysis

For the primary analysis (reported elsewhere) [8], we required 80 LBP participants in order to detect a 15% change over time in PPT (effect size 0.64) at the lumbar spine, comparing two groups with 80% power (alpha 0.05) [43, 44]. This sample size is adequate to compare change over time between episodic and persistent LBP groups using repeated measures in this manuscript. We anticipated unequal group sizes due to our inability to control the number of episodic and persistent LBP participants that would enrol. For the non-LBP participants, a total sample size of 22 (*n* = 11 per group) had 80% power (alpha 0.05) to detect an effect size difference of 0.64 (retained from previous calculation) between two groups (G*Power v3.1.9.4, University of Düsseldorf, Germany). Given that PPT has shown wide variability in other studies [16, 18, 45], we conservatively planned for a minimum of 20 participants per group. This is also in line with numerous other studies comparing differences between groups in QST [16, 45, 46]. Our final sample consisted of *n* = 20 non-LBP, *n* = 23 episodic LBP, and *n* = 57 persistent LBP.

We present continuous descriptive data using means and standard deviations or medians and interquartile ranges, and ranges. For categorical data, we report frequency distributions. Graphical inspection revealed that the PPT and TS data were left-skewed with several outliers.

Potential modifying variables were tested using univariate linear regression models, including age, sex, pain catastrophising, anxiety, baseline subjective LBP intensity, and intervention group. Intervention group was not included as a covariate since it was not relevant in the univariate models, there were no meaningful differences in change in QST between intervention groups [8], and the episodic and persistent LBP trajectory groups had a similar distribution of participants that received SMT and sham. Baseline subjective LBP intensity was not relevant based on univariate models and was not a statistically significant modifier when included as a fixed effect in the models. Generalised linear mixed models with log link and linear mixed models were used to analyse longitudinal PPT and TS data between trajectory groups. The final models included random intercept subject effects, random slope time effects, and sex and age as fixed effects. Baseline differences between non-LBP, episodic LBP, and persistent LBP trajectories were analysed using pairwise comparisons in the longitudinal models. As mixed models use maximum likelihood estimation to estimate parameters, all participants were included in analyses, irrespective of missing data.

PPT and TS outcome data are summarised using adjusted marginal means and their 95% confidence intervals. Stata/IC v15.1 (StataCorp LP, College Station, USA) was used for all analyses. All hypotheses were 2-sided, and *p* values of <.05 were considered statistically significant.

## Results

**Fig. 1:**
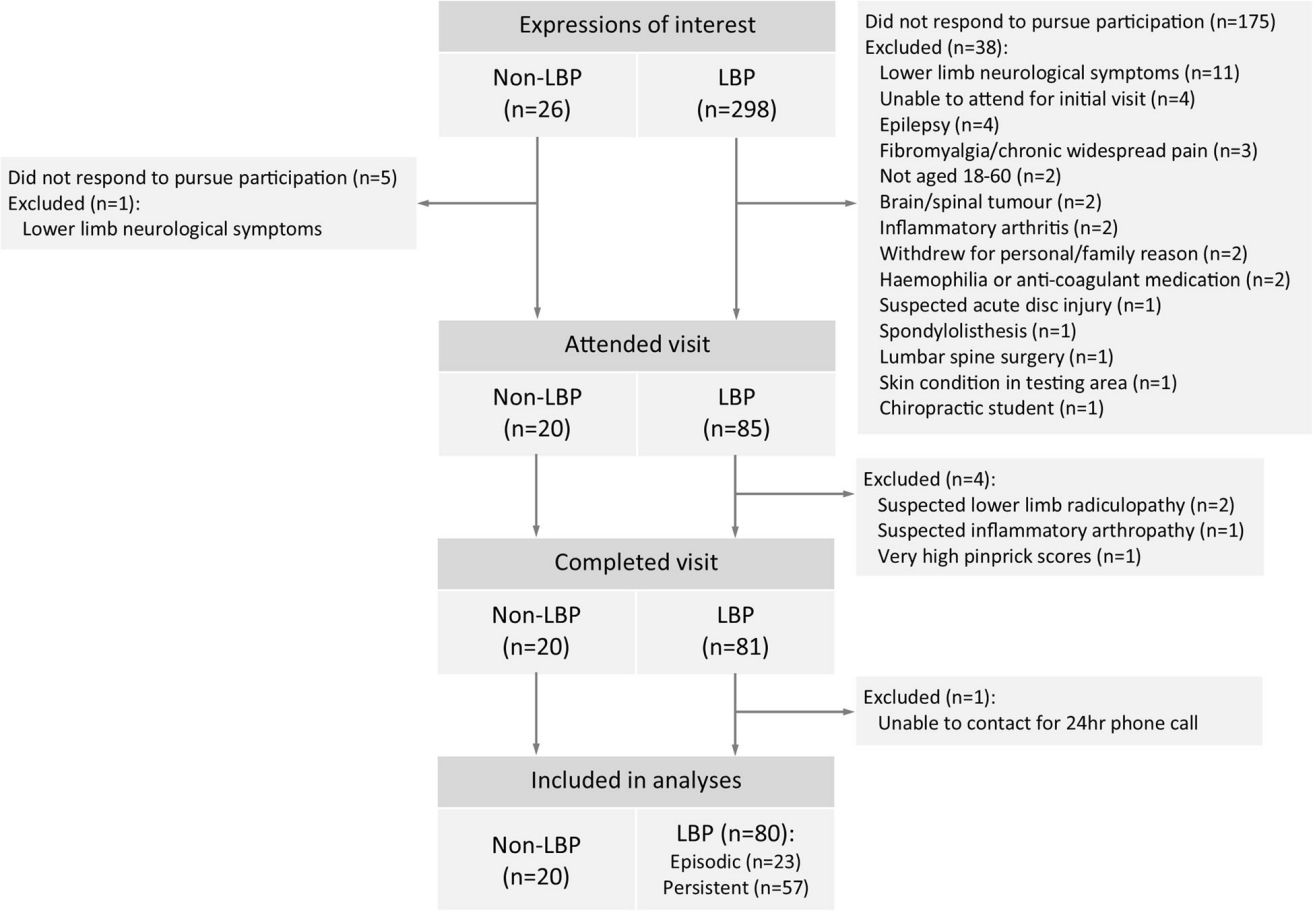
Participant flow chart. Abbreviations: LBP = low back pain

A total of 101 individuals participated in the study from Oct 2017 to July 2018. One participant with LBP was excluded from analyses as they were uncontactable for the 24 h follow-up call, leaving a total of 20 non-LBP participants, 23 episodic LBP participants, and 57 persistent LBP participants. See Fig. 1 for a participant flow chart. Due to computer error some PPT data for some LBP participants were not recorded. Specifically, baseline PPT data were missing for two participants, and 30 min PPT data were missing for three other participants. We did not impute this data since mixed model analyses can be run despite missing data. Six participants had some individual PPT measures missing (e.g. third round of testing at 15 min). We imputed these missing data by using the measurement that was recorded at that time point (e.g. second round at 15 min).

Nine instances of harms were reported during the study, all of which resolved within several days and we considered to be common side effects [47]: six participants who received SMT and three participants who received a sham reported increased LBP or post-treatment soreness after participating, four of whom also reported thigh pain.

### Participant characteristics

All three trajectory groups were similar in age. There were no differences in the duration of the LBP problem (taken from the subjective history), or in intervention group allocation and intervention guess in the LBP groups. The non-LBP group had a somewhat higher proportion of male participants than the LBP groups. Both LBP groups had higher pain catastrophising than the non-LBP group, the persistent LBP trajectory group had higher anxiety than the non-LBP group, and the persistent LBP group had higher LBP intensity than the episodic LBP group. There were four LBP participants, all with episodic LBP, who reported no subjective LBP in the 24 h prior to participating. See Table 1 for baseline participant characteristics.

**Table 1:** Baseline participant characteristics

	Non-LBP (*n* = 20)	Episodic LBP (*n* = 23)	Persistent LBP (*n* = 57)
**Age in years, mean (SD, range)**	31 (SD 11, 19–57)	35 (SD 13, 18–59)	37 (SD 12, 18–58)
**Sex**	7 female (35%), 13 male (65%)	14 female (61%), 9 male (39%)	28 female (49%), 29 male (51%)
**LBP intensity on 0–10 NRS, median (IQR, range)**			
**Current LBP**	–	1.0 (IQR 2.0, 0–4)	3.0 (IQR 3.0, 0–7)
**Average LBP in previous 24 h**	–	2.0 (IQR 3.0, 0–6)	5.0 (IQR 2.5, 1–8)
**Worst LBP in previous 24 h**	–	3.0 (IQR 4.0, 0–7)	6.0 (IQR 2.5, 1–10)
**Best LBP in previous 24 h**	–	0.0 (IQR 1.0, 0–2)	1.0 (IQR 3.0, 0–7)
**Duration of LBP problems**	–	≥10 years: 10 (43.5%)	≥10 years: 25 (43.9%)
		3–9 years: 7 (30.4%)	3–9 years: 17 (29.8%)
		1–2 years: 4 (17.4%)	1–2 years: 13 (22.8%)
		3–11 months: 2 (8.7%)	3–11 months: 2 (3.5%)
**Pain Catastrophizing Scale score (0–52), mean (SD, range)**	6.3 (SD 6.8, 0–26)	13.4 (SD 9.8, 0–40)	14.2 (SD 9.5, 0–40)
**PROMIS Anxiety T-score, mean (SD, range)**	49 (SD 8.6, 39.1–71.3)	51.7 (SD 9.2, 39.1–71.3)	54.5 (SD 8.7, 39.1–74.1)
**Intervention group**	–	12 SMT (52%), 11 sham (48%)	28 SMT (49%), 29 sham (51%)
**Guess for intervention group**	–	18 real (78%), 5 not real (22%)	40 real (70%), 17 not real (30%)
**Calf PPT (kg/cm**^**2**^**), median (IQR)**	4.0 (IQR 3.7)	3.5 (IQR 3.3)	3.8 (IQR 3.6)
**Lumbar PPT (kg/cm**^**2**^**), median (IQR)**	5.5 (IQR 4.1)	4.1 (IQR 4.6)	4.4 (IQR 4.6)
**Shoulder PPT (kg/cm**^**2**^**), median (IQR)**	3.1 (IQR 2.6)	2.3 (IQR 2.3)	2.6 (IQR 2.4)
**Hand TS (0–100 NRS), median (IQR)**	2.6 (IQR 13.2)	5.0 (IQR 13.7)	5.2 (IQR 9.5)
**Feet TS (0–100 NRS), median (IQR)**	10.9 (IQR 20.1)	8.3 (IQR 19.7)	10.8 (IQR 14.7)

*Abbreviations*: *IQR* interquartile range, *LBP* low back pain, *NRS* numerical rating scale, *PPT* pressure pain threshold, *SD* standard deviation, *SMT* spinal manipulative therapy, *TS* temporal summation

### Research questions

**Fig. 2:**
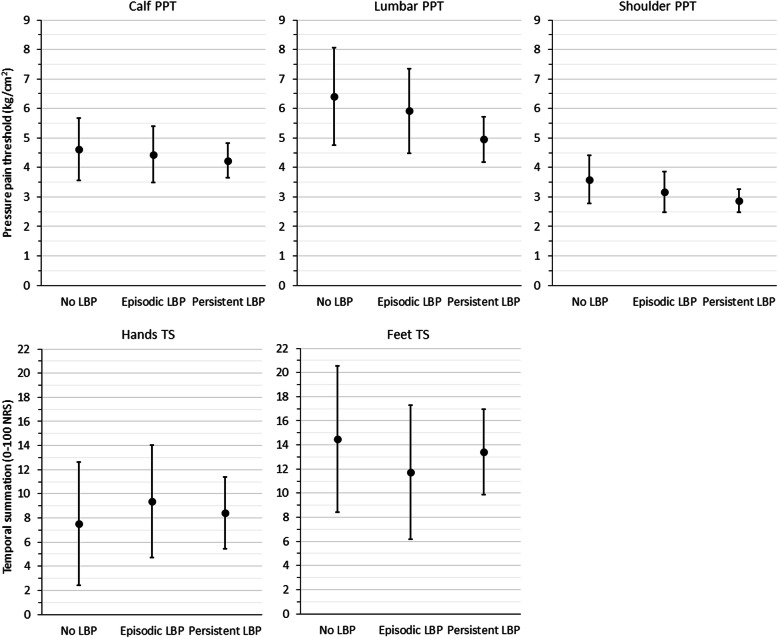
Adjusted marginal means and 95% confidence intervals for baseline pressure pain threshold and temporal summation by low back pain trajectory. Abbreviations: LBP = low back pain, NRS = numerical rating scale, PPT = pressure pain threshold, TS = temporal summation

There were no significant differences between the non-LBP group and the episodic or persistent LBP groups in baseline PPT or TS at any testing site. See Table 2 and Fig. 2 for full between-group baseline PPT and TS results.

**Table 2:** Adjusted marginal means for baseline pressure pain threshold and temporal summation by low back pain trajectory

	Adjusted marginal means (95% CI)			Between-group difference *p* value	
	Non-LBP	Episodic LBP	Persistent LBP	Non-LBP vs. episodic LBP	Non-LBP vs. persistent LBP
**Calf PPT (kg/cm**^**2**^**)**	4.61 (3.55–5.68)	4.44 (3.48–5.40)	4.23 (3.65–4.82)	0.808	0.532
**Lumbar PPT (kg/cm**^**2**^**)**	6.41 (4.75–8.07)	5.92 (4.48–7.36)	4.95 (4.17–5.72)	0.660	0.111
**Shoulder PPT (kg/cm**^**2**^**)**	3.59 (2.78–4.40)	3.17 (2.49–3.85)	2.88 (2.48–3.27)	0.429	0.115
**Hand TS (0–100 NRS)**	7.53 (2.43–12.64)	9.40 (4.74–14.06)	8.42 (5.47–11.38)	0.599	0.768
**Feet TS (0–100 NRS)**	14.49 (8.43–20.54)	11.74 (6.17–17.30)	13.42 (9.90–16.95)	0.513	0.767

*Abbreviations*: *LBP* low back pain, *PPT* pressure pain threshold, *TS* temporal summation, *NRS* numerical rating scale

**Fig. 3:**
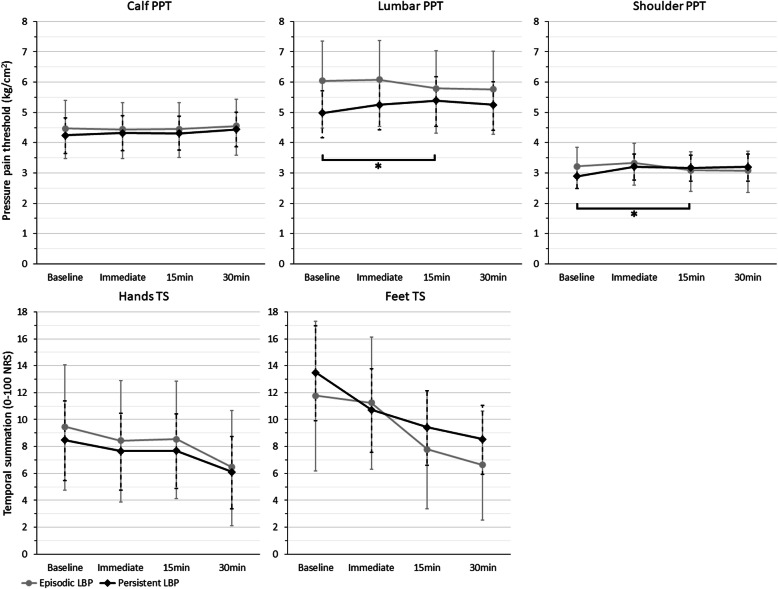
Adjusted mean change and 95% confidence intervals for pressure pain threshold and temporal summation after lumbar manual therapy intervention, by low back pain trajectory. * *p* < .05 for between-group differences over time (time by trajectory interaction). Abbreviations: LBP = low back pain, NRS = numerical rating scale, PPT = pressure pain threshold, TS = temporal summation

There were significant differences in the change in lumbar and shoulder PPT from baseline to 15 min, when comparing the episodic and persistent LBP trajectories. At both sites, the persistent LBP group had an increase in PPT from baseline to 15 min, while the episodic LBP group had a decrease. There were no other significant differences over time based on LBP trajectory. See Table 3 and Fig. 3.

**Table 3:** Differences between low back pain trajectories in change of pressure pain threshold and temporal summation after lumbar manual therapy intervention

Testing site and time	Adjusted marginal means (95% CI)		Between-group (time x trajectory) ***p*** value
	Episodic LBP	Persistent LBP	
**Calf PPT (kg/cm**^**2**^**)**			
Baseline	4.44 (3.48–5.40)	4.23 (3.65–4.82)	–
Immediate	4.40 (3.48–5.32)	4.31 (3.74–4.89)	.537
15 min	4.42 (3.51–5.32)	4.31 (3.75–4.87)	.663
30 min	4.51 (3.59–5.44)	4.43 (3.86–5.01)	.652
**Lumbar PPT (kg/cm**^**2**^**)**			
Baseline	5.92 (4.48–7.36)	4.95 (4.17–5.72)	–
Immediate	5.96 (4.54–7.38)	5.22 (4.42–6.02)	.261
15 min	5.68 (4.32–7.03)	5.35 (4.54–6.17)	.025*
30 min	5.65 (4.28–7.02)	5.21 (4.40–6.02)	.157
**Shoulder PPT (kg/cm**^**2**^**)**			
Baseline	3.17 (2.49–3.85)	2.88 (2.48–3.27)	–
Immediate	3.28 (2.59–3.97)	3.20 (2.77–3.63)	.128
15 min	3.04 (2.39–3.69)	3.15 (2.72–3.58)	.026*
30 min	3.04 (2.36–3.71)	3.18 (2.73–3.62)	.058
**Hands TS (0–100)**			
Baseline	9.40 (4.74–14.06)	8.42 (5.47–11.38)	–
Immediate	8.38 (3.88–12.88)	7.60 (4.75–10.45)	.887
15 min	8.49 (4.12–12.86)	7.64 (4.87–10.40)	.934
30 min	6.41 (2.13–10.69)	6.05 (3.35–8.76)	.691
**Feet TS (0–100)**			
Baseline	11.74 (6.17–17.30)	13.42 (9.90–16.95)	–
Immediate	11.21 (6.30–16.13)	10.66 (7.55–13.77)	.175
15 min	7.75 (3.36–12.14)	9.37 (6.59–12.15)	.971
30 min	6.59 (2.54–10.64)	8.49 (5.92–11.05)	.931

*Abbreviations*: *LBP* low back pain, *PPT* pressure pain threshold, *TS* temporal summation

^*^p < .05

## Discussion

### Summary

We found no significant differences in baseline PPT and TS between people without LBP and those with episodic or persistent LBP. However, we noted a consistent pattern of lower baseline PPT (higher sensitivity) across the three trajectories, from non-LBP to persistent LBP, which was strongest at the lumbar spine. Based on participant’s pattern of LBP, there was no difference in how PPT and TS change over time after lumbar SMT and sham.

### Explanation and comparisons

The non-significant pattern noted above aligns with expectations, based on prior research, that PPT is lower in chronic LBP compared to asymptomatic populations. The variability in PPT in this study (particularly the non-LBP and episodic LBP groups) is similar to that observed in other studies [18, 26, 45, 48]. The large variability may also be a symptom of heterogeneity in the trajectory groups. Since participants were categorised based on self-reported LBP over the previous 12 months, the trajectory grouping does not account for recent pain frequency, intensity, or disability, and therefore the LBP experiences of participants within the episodic and persistent groups may vary widely. Thus, there may be actual differences between LBP trajectory groups that are masked by the large variability in PPT. It is also possible, however, that there are no real differences in PPT between LBP trajectory groups. If there is in fact a real difference between trajectory groups, a larger sample size may be required to confirm this. Other types of QST may also offer additional insight, and it remains unclear which individual QST procedures are most clinically relevant for LBP. Composite QST scores [49] may offer a more clinically relevant approach to this problem. It is possible that relevant group differences in QST have been overlooked in the current study due to the limitations in our QST protocol, including our TS protocol which differs somewhat from the widely used DFNS protocol [41].

Our results agree with the only other study that has investigated a recurrent LBP population, which found there were no differences between healthy controls, people with recurrent LBP, mild chronic LBP, or severe chronic LBP in local or remote PPT and TS [27]. The participants in our persistent LBP trajectory group would typically be considered to have chronic LBP, thus we expected that they would have comparable QST outcomes to other studies in chronic LBP populations. However, the results of both our study and Goubert et al. [27] contradict the recent systematic review [13] which concluded in favour of decreased remote PPTs and increased local TS (but not hand TS) in sub-acute and chronic LBP populations compared to healthy controls. Our results also contradict the numerous studies showing locally decreased PPT in chronic LBP populations compared to healthy controls [16–26].

On comparing populations, participants in other studies were similar [18, 19] or older [17, 20, 22, 45] in age and had similar [19, 20, 22, 45] or higher [17, 18] pain intensity. Our study recruited from the general population, compared to other studies which recruit from primary care [17, 18, 21], secondary care [45], and specialist pain centres [19, 20, 22]. The primary purpose of our trial involved delivering a LBP intervention, and LBP participants were offered some ‘free’ pragmatic chiropractic treatment for LBP after participating as an incentive. Hence, our participants likely had some level of motivation for seeking ‘care’ by participating in the study. Unfortunately, we did not collect data on disability to enhance this comparison. On the whole it appears that our LBP participants were younger and perhaps had more ‘mild’ LBP compared to most other studies, and are recruited from a different population. These differences may have resulted in smaller or absent differences between groups. It is also possible that the differences may relate to the specific testing sites chosen, or to other differences in the populations. Since we only measured remote TS, we would have missed potential local differences in TS.

For the above reasons, the clinical relevance of our findings is unclear, and, especially given that our results contradict the bulk of the literature, should be interpreted with caution and while considering the limitations of this study. Further investigation of differences in QST between LBP trajectories in various populations, and with an expanded QST protocol, is likely worthwhile and will contribute to an understanding of the sensitisation processes occurring in these subgroups.

Based on our data, it does not appear that a participant’s LBP trajectory affects how PPT and TS change after lumbar SMT and sham. We are not aware of any other studies comparing how QST changes over time after manual therapy intervention between different LBP trajectories, and thus cannot make any comparisons. It is worth noting that these are experimental outcomes and our results do not imply any conclusions regarding the clinical value of SMT for LBP. We also note that in the primary analysis of this data there were no differences in change in QST after lumbar SMT compared to sham [8]. Hypoalgesia in PPT did not occur at all in either group, while hypoalgesia in TS occurred equally in both groups [8]. Recent systematic reviews have observed that there might be differences in manipulation-induced hypoalgesia based on where the SMT is applied [29, 50]. Since studies often show no change in PPT after lumbar SMT in clinical populations [29], it may be that the question of whether a participant’s pain trajectory acts as a modifier of manipulation-induced hypoalgesia is irrelevant in this population (at least regarding PPT). This topic may be worth pursuing in other regions of the spine.

### Methodological considerations

The persistent LBP group had significantly higher baseline pain intensity and higher anxiety than the episodic LBP group, supporting our assumption that these were distinct clinical groups. Our criteria that non-LBP participants must not have had bothersome LBP for 1 year prior to participating allows us to be confident that this group is fairly representative of a “non-LBP” trajectory, as it would be unlikely to capture people with episodic LBP who happened to be asymptomatic at the time of recruitment.

It is possible that LBP participants who are not in pain at the time of participating may have different QST outcomes than those who are in pain. We did not control for this statistically for several reasons. Univariate analyses indicated subjective pain intensity was not a relevant modifier for our data, and we had only four participants who reported no pain in the 24 h prior to participating. There is also typically poor correlation between PPT or TS and subjective pain intensity [19, 51].

We categorised participants based on their self-reported LBP trajectory over the previous year, and we recognise that this introduces some error in that participants may have selected an inappropriate trajectory, and that the trajectories questionnaire is subject to recall bias. However, the questionnaire has shown acceptable criterion validity compared to trajectories derived from 6-month frequent data collection [34]. We also recognise that grouping LBP participants into episodic and persistent trajectories squanders some of the detail available if we had used more specific trajectories, and that the questionnaire was not necessarily designed to be collapsed in this manner. However, this is the first study on this topic that we are aware of, and we would have had insufficient power to analyse the data based on detailed trajectories. Therefore, we decided that this was an acceptable starting point. For the episodic group, we also note that the questionnaire does not control for the duration of LBP episodes or whether it was a first ever episode, though only two episodic LBP participants reported having their first experience of LBP within 3–11 months of participating in the study (none were less than this).

Measuring other types of QST may have offered additional insight into this topic, but we chose to measure only PPT and TS as they were the most relevant QST types for the planned primary analysis of this data, published elsewhere [8]. It should also be noted that our protocol for TS testing differs somewhat from the DFNS protocol [41], though the protocol was pre-tested prior to this study [42].

Finally, it may be considered a limitation that SMT was delivered to a pre-specified segment of the spine rather than to a ‘dysfunctional’ segment, as would typically occur in clinical practice. This decision was made in order to improve standardisation and repeatability, and acknowledging that manipulation-induced hypoalgesia appears to occur regardless of whether a standardised or pragmatic approach to the treatment target is taken [52].

## Conclusion

There were no statistically significant differences between LBP trajectory subgroups (no LBP, episodic LBP, and persistent LBP) in baseline PPT or TS. These findings contradict the literature which consistently demonstrates hyperalgesia in chronic LBP populations. This may reflect differences in our approach to subgrouping (trajectories compared to traditional acute, subacute, and chronic), differences in our population, or limitations to our QST protocol. We did, however, observe a consistent pattern of decreased baseline PPT in the episodic and persistent LBP groups compared to those without LBP, which is consistent with the literature. Thus, in spite of our statistically non-significant findings, baseline differences in QST between LBP trajectories may be worth further research. We found that short term changes in PPT and TS after a brief manual therapy intervention did not differ between those with an episodic or persistent LBP trajectory.

## Data Availability

The datasets analysed during the current study are available from the corresponding author on reasonable request.

## Abbreviations

LBP: Low back pain
PPT: Pressure pain threshold
QST: Quantitative sensory testing
SMT: Spinal manipulative therapy
TS: Temporal summation
